# Ti_3_C_2_ MXene-Coated Germanium Nanoparticles on Nickel Foam for Binder-Free Lithium-Ion Battery Anodes

**DOI:** 10.3390/nano16150969

**Published:** 2026-08-06

**Authors:** Junaid Aslam, Muhammad Arif Khan, Weiwei Sun, Chao Yang

**Affiliations:** 1 School of Environmental and Chemical Engineering, Shanghai University, Shanghai 200444, China; junaidaslam@shu.edu.cn; 2 Department of Physics, College of Sciences, Shanghai University, Shanghai 200444, China; muhammad666@shu.edu.cn

**Keywords:** anode, germanium, lithium-ion batteries, MXene, nickel foam

## Abstract

Germanium (Ge) is a promising high-capacity anode material for lithium-ion batteries; however, its practical application remains limited by substantial volume variation, unstable interfacial reactions, and rapid capacity degradation during repeated lithiation/delithiation. In this work, a binder-free Ge/C/MXene@NF hybrid electrode was developed through a sequential fabrication process, where Ge nanoparticles were immobilized within a PVP-derived carbon matrix supported on a three-dimensional nickel-foam scaffold and subsequently integrated with a Ti_3_C_2_T_x_ MXene conductive network to construct a hierarchical Ge/C/MXene hybrid architecture. The nickel foam provides a continuous current-collecting framework and mechanical support, while the MXene network improves electrical connectivity, electrolyte accessibility, and interfacial charge-transfer kinetics. Structural and compositional analyses further indicate the presence of PVP-derived carbon and a possible minor NiGe interfacial phase formed during annealing. Comparison with Ge@NF and the individual component electrodes provides insight into the respective contributions of MXene, Ge, and the PVP-derived carbon framework to the electrochemical behaviour of the composite electrode. Using the total deposited active-material mass as the normalisation basis, the MXene@Ge@NF electrode retains a reversible specific capacity of 789.8 mAh g^−1^ after 100 cycles at an effective current density of 76.2 mA g^−1^. The observed electrochemical behaviour originates from the integrated contributions of Ge nanoparticles, the PVP-derived carbon matrix, the conductive Ti_3_C_2_T_x_ MXene network, the three-dimensional nickel-foam scaffold, and possible Ni–Ge interfacial interactions. Rather than representing a Ge-dominated electrode, this architecture demonstrates the advantages of integrating multiple functional components within a binder-free Ge/C/MXene hybrid architecture.

## 1. Introduction

Lithium-ion batteries (LIBs) have become indispensable energy-storage systems for portable electronics, electric vehicles, and grid-scale applications owing to their high energy density, long cycle life, and mature manufacturing technology [1,2]. Commercial LIBs commonly employ graphite as the anode material because of its low working potential, good cycling stability, natural abundance, and relatively low cost [3]. However, the limited theoretical specific capacity of graphite, 372 mAh g^−1^, restricts further improvement in cell-level energy density and makes it increasingly difficult to satisfy the growing demand for high-energy and fast-charging batteries [4]. Therefore, considerable effort has been devoted to developing alternative anode materials with higher reversible capacities and improved rate capability. Among them, germanium (Ge), a group IV element, has attracted significant attention as a high-capacity alloy-type anode for LIBs [5]. Although the theoretical capacity of Ge, approximately 1600 mAh g^−1^, is lower than that of silicon, Ge offers several kinetic advantages, including faster Li-ion diffusivity and higher intrinsic electrical conductivity [6]. These properties are beneficial for improving charge-transfer kinetics and rate performance. Nevertheless, similar to other alloy-type anodes, Ge undergoes severe volume variation during repeated lithiation/delithiation, leading to unstable electrode/electrolyte interfaces, and rapid capacity decay [7]. To address these challenges, rational nanostructure design has been widely explored as an effective strategy to accommodate volume expansion, shorten ion-diffusion pathways, and enhance the structural stability of Ge-based anodes [8]. Among these approaches, nanostructure engineering has attracted particular attention because it provides an effective route to balance the high theoretical capacity of Ge with improved mechanical resilience and electrochemical reversibility.

Considerable research effort has therefore been directed toward improving the structural durability and electrochemical reversibility of Ge anodes [9,10,11]. Rational nanostructuring and composite engineering are among the most effective approaches for mitigating volume-induced degradation and enhancing lithium-storage kinetics [12]. Ge nanostructures, including nanoparticles, nanosheets, and nanowires, have been explored because their reduced dimensions can shorten Li-ion diffusion pathways, increase active interfaces, and partially relieve mechanical stress during cycling [13,14,15]. In addition, conductive carbonaceous matrices and two-dimensional frameworks have been incorporated into Ge-based electrodes to improve electrical conductivity, stabilize the electrode/electrolyte interface, and buffer the strain generated by Ge volume variation [16,17]. Although these strategies have achieved notable progress, maintaining intimate electrical contact, robust structural integrity, and efficient ion/electron transport simultaneously remains a critical challenge for high-capacity Ge-based anodes [18,19]. Among various two-dimensional materials, MXenes have emerged as attractive components for advanced energy-storage electrodes because of their high electrical conductivity, tunable surface chemistry, hydrophilic characteristics, and layered structure [20,21]. Ti_3_C_2_T_x_, a representative MXene, is particularly suitable for constructing conductive interfacial networks, as its surface terminations and layered morphology can facilitate charge transport and improve electrode/electrolyte contact [22]. Compared with conventional graphene-based materials, Ti_3_C_2_T_x_ may offer favorable Li-ion transport kinetics because of its lower diffusion barrier and accessible surface chemistry [23]. Previous studies have also shown that Ti_3_C_2_T_x_ -based composites can enhance charge-transfer behavior and help alleviate the mechanical instability of high-capacity alloy-type anodes [24,25]. Therefore, integrating Ti_3_C_2_ MXene with Ge-based electrodes offers a promising route to construct conductive and mechanically supportive interfaces for improved lithium-storage performance.

In this work, a binder-free MXene-integrated Ge/C@NF hybrid electrode was constructed through a sequential assembly strategy, where Ge nanoparticles were immobilized within a PVP-derived carbon matrix supported on a three-dimensional nickel-foam scaffold, followed by the integration of a Ti_3_C_2_T_x_ MXene conductive network. This architecture was designed to address the major limitations associated with Ge-based anodes, including volume-induced structural degradation, unstable interfacial contact, and limited charge-transfer pathways during repeated lithiation/delithiation. Within this integrated electrode framework, Ge nanoparticles provide alloying-based lithium-storage capability, while the PVP-derived carbon matrix contributes to electrical connectivity, structural buffering, and interfacial stabilization. Meanwhile, the Ti_3_C_2_T_x_ MXene network facilitates electron transport and electrolyte accessibility, and the three-dimensional nickel foam scaffold provides continuous current collection and mechanical support. A possible minor Ni–Ge interfacial phase formed during annealing may further influence the interfacial contact between the deposited active components and the nickel substrate. Therefore, the electrochemical response of the electrode is considered to originate from the integrated functions of the Ge/C/MXene composite architecture rather than from the contribution of an individual component alone. Accordingly, the objective of this study was to fabricate and systematically evaluate a sequentially assembled, three-dimensional MXene-integrated Ge/C@NF electrode using direct comparisons with Ge@NF and individual component electrodes.

Accordingly, the objective of this study was to fabricate and systematically evaluate a sequentially assembled, three-dimensional MXene-integrated Ge/C@NF electrode (MXene@Ge@NF) using direct comparisons with Ge@NF and individual component electrodes. Recent studies have already demonstrated Ge/MXene composites and three-dimensional Ge electrodes supported on conductive metal frameworks. Therefore, the present work is not positioned as the first example of such an electrode architecture. Instead, this study systematically evaluates a sequentially fabricated, binder-free MXene@Ge@NF electrode in which Ge nanoparticles are immobilised on a three-dimensional nickel-foam scaffold and subsequently covered by a conductive Ti_3_C_2_T_x_ MXene network. Comparison with Ge@NF and the individual component electrodes was performed to evaluate the individual roles of Ge, Ti_3_C_2_T_x_ MXene, and the PVP-derived carbon matrix in determining the electrochemical behaviour of the composite electrode. The observed behaviour is attributed to the combined contributions of crystalline Ge, the MXene network, PVP-derived carbon, the nickel-foam framework, and a possible minor NiGe interfacial phase. Accordingly, the scientific contribution of this work lies in the systematic evaluation of the integrated electrode and its component functions using clearly defined gravimetric and areal performance metrics, rather than in claiming novelty solely from the spatial arrangement of the constituent materials.

To further contextualize the contribution of this work within recent developments in Ge-based lithium-ion battery anodes, a comparison with representative reported Ge-based electrode systems is provided in Table 1. The selected studies include carbon-supported Ge composites, graphene-assisted Ge electrodes, Ge-based conversion materials, and directly supported/self-supported Ge architectures. The comparison highlights the influence of electrode architecture, conductive framework, binder-free design, and structural integration on the electrochemical behaviour of Ge-based electrodes. Unlike conventional slurry-cast composite electrodes, the present MXene@Ge@NF architecture integrates Ge nanoparticles, PVP-derived carbon, Ti_3_C_2_T_x_ MXene, and a three-dimensional nickel-foam scaffold into a binder-free electrode configuration. This comparison provides a broader context for evaluating the design strategy of the present work and highlights the importance of integrated electrode architecture, structural support, and transparent performance evaluation in developing advanced Ge-based anodes.

## 2. Materials and Methods

### 2.1. Materials

Titanium aluminum carbide (Ti_3_AlC_2_, 99%), lithium fluoride (LiF), hydrochloric acid (HCl, 37 wt%, 95% purity), potassium permanganate (KMnO_4_), hydrogen peroxide (H_2_O_2_), hydrazine hydrate (N_2_H_4_·H_2_O), toluene (C_7_H_8_), polyvinylpyrrolidone (PVP, (C_6_H_9_NO)n), N-methyl-2-pyrrolidone (NMP, C_5_H_9_NO), and copper foil (Cu, 99.9%) were purchased from Sigma-Aldrich, Germany (St. Louis, Missouri, MO, USA). (Ge, ~30 nm) were purchased from Shanghai ST-Nano Science and Technology Co., Ltd. (Shanghai, China). Lithium hexafluorophosphate (LiPF_6_), ethylene carbonate (EC), diethyl carbonate (DEC), and ethyl methyl carbonate (EMC) were purchased from Aladdin (Shanghai, China). Lithium hexafluorophosphate (LiPF_6_), ethylene carbonate (EC), diethyl carbonate (DEC), and ethyl methyl carbonate (EMC) were purchased from Aladdin (Shanghai, China). The electrolyte solution was prepared by dissolving 1 M LiPF_6_ in a mixed solvent of EC, DEC, and EMC with a volume ratio of 1:1:1. Celgard 2320 polypropylene separator was purchased from Celgard LLC (Charlotte, NC, USA). X-ray diffraction (XRD) patterns were analyzed using MDI Jade software (version 6.5; Materials Data Inc., Livermore, CA, USA), while electrochemical data processing, graphical representation, and statistical analysis were performed using OriginPro 2024 (version 10.1; OriginLab Corporation, Northampton, MA, USA).

### 2.2. Sample Preparation

Ti_3_C_2_Tx MXene nanosheets were deposited onto the pre-formed Ge@NF scaffold. In a typical procedure, 1 g of LiF was slowly added to 20 mL of 9 M HCl under continuous stirring. Subsequently, 1 g of Ti_3_AlC_2_ powder was gradually introduced into the solution. The reaction mixture was maintained at 35 °C for 48 h under continuous magnetic stirring to promote the effective selective etching of Al from Ti_3_AlC_2_ and the formation of multilayer Ti_3_C_2_T_x_. The obtained sediment was redispersed in water and gently homogenized to form a uniform suspension, followed by centrifugation at 1800 rpm for 30 min to obtain few-layer MXene nanosheets. Nickel foam was ultrasonically cleaned in ethanol and deionized water for 15 min each to remove surface impurities and improve wettability. A suspension containing 0.09 g of Ge nanoparticles (~30 nm) and 0.1 g of polyvinylpyrrolidone (PVP) was prepared in deionized water and stirred for 2 h at room temperature to achieve uniform dispersion. The cleaned nickel foam was then immersed in the Ge/PVP suspension for 30 min to allow sufficient adsorption of nanoparticles onto the 3D conductive scaffold. The sample was subsequently dried in a vacuum oven at 80 °C for 12 h. The dried composite was then thermally treated at 850 °C for 2 h under an argon atmosphere with a heating rate of 5 °C min^−1^. During this process, PVP decomposed to form a conductive carbon layer, which enhanced interfacial adhesion and electrical conductivity while firmly anchoring Ge nanoparticles onto the nickel foam framework. The MXene@Ge@NF composite electrode was fabricated using a repeated immersion–drying process, which was performed three times to obtain a homogeneous and conformal MXene coating over the Ge@NF framework. The Ge@NF substrate was immersed in a Ti_3_C_2_Tx MXene aqueous dispersion (2 mg mL^−1^) for 10 min at room temperature to ensure uniform infiltration of MXene nanosheets into the porous structure. The electrode was then dried at 60 °C for 12 h under vacuum conditions. 

**Figure 1:**
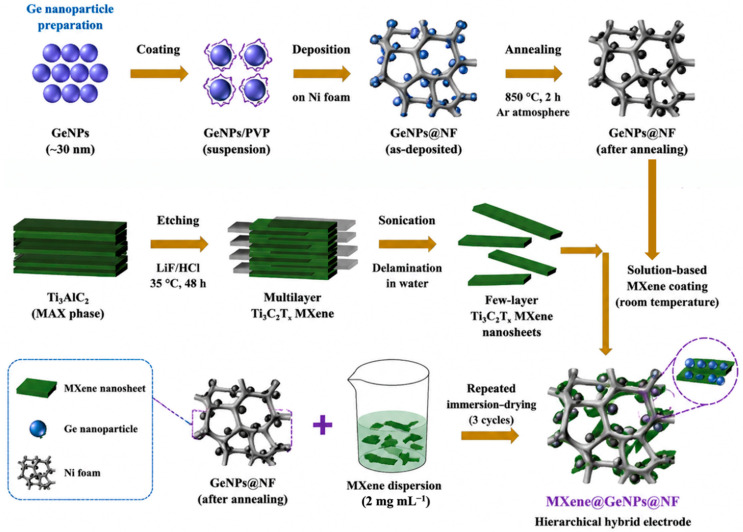
Schematic diagram of the fabrication process of MXene@Ge@NF composite.

The active-material loading of the final MXene@Ge@NF electrode was determined through sequential gravimetric measurements. The nickel foam substrate had a diameter of 12 mm and a geometric area of 1.131 cm^2^. After Ge/PVP deposition and thermal treatment at 850 °C under Ar, the mass of the annealed Ge/PVP-derived carbon intermediate was 1.116 mg. Following MXene coating and final drying, the total deposited active-material mass increased to 1.464 mg, corresponding to an active-material loading of 1.294 mg cm^−2^. The additional mass increase after MXene incorporation confirmed the deposition of Ti_3_C_2_T_x_ MXene nanosheets onto the Ge/PVP-derived carbon framework. Considering the carbonaceous species generated from PVP decomposition and the MXene-derived inorganic components may undergo complex transformations during thermal treatment and electrochemical operation, the individual component fractions were regarded as estimated contributions rather than absolute compositional values. Therefore, the gravimetric measurements were mainly used to determine the total deposited active-material mass and to establish a consistent basis for electrochemical performance normalization. The nickel foam substrate was excluded from these active-material calculations. The resulting electrode was considered as an integrated Ge/C/MXene composite architecture, in which each component contributes to the overall electrochemical response. The overall fabrication route of the MXene@Ge@NF electrode is schematically illustrated in Figure 1. The process involves the deposition of the Ge/PVP suspension onto the three-dimensional nickel-foam framework, thermal treatment under Ar to form the annealed Ge/PVP-derived carbon intermediate, and subsequent deposition of Ti_3_C_2_T_x_ MXene through repeated immersion and drying. This sequential process produces an integrated, binder-free electrode in which the deposited active layer is directly supported on the nickel-foam scaffold. Figure S1 further provided a photographic record of the corresponding experimental stages. The cleaned nickel foam was first coated with the Ge/PVP suspension and subsequently annealed under Ar to produce the Ge/PVP-derived carbon intermediate. The annealed intermediate was then repeatedly immersed in the Ti_3_C_2_T_x_ MXene dispersion and dried to obtain the final MXene@Ge@NF electrode. The progressive darkening of the nickel-foam surface provides qualitative visual evidence of the successive deposition and thermal-treatment steps.

### 2.3. Material Characterization

X-ray diffraction (XRD) was used to analyze the crystal structure of the samples (instrument model: Hitachi/Rigaku X-ray diffraction system (Hitachi High-Tech Corporation/Rigaku Corporation, Tokyo, Japan). X-ray diffraction (XRD) was performed to analyze the crystal structures of the samples using a Hitachi/Rigaku X-ray diffraction system (Hitachi High-Tech Corporation/Rigaku Corporation, Tokyo, Japan). The morphology and microstructure of the materials were examined by transmission electron microscopy (TEM, JEM-series, JEOL Ltd., Tokyo, Japan) and scanning electron microscopy (SEM, JSM-series, JEOL Ltd., Tokyo, Japan). Energy-dispersive X-ray spectroscopy (EDS, Oxford Instruments, Oxford, UK) was employed to investigate the elemental distribution within the nanocomposites. Raman spectroscopy was conducted using a Renishaw in Via Raman system (Renishaw plc, Wotton-under-Edge, UK) to evaluate structural defects and graphitization characteristics. Thermogravimetric analysis (TGA, NETZSCH STA 409, NETZSCH-Gerätebau GmbH, Selb, Germany) was carried out under a nitrogen (N_2_) atmosphere from approximately 30 °C to 800 °C at a heating rate of 10 °C min^−1^ to assess thermal stability. Fourier-transform infrared spectroscopy (FTIR, Thermo Scientific Nicolet iS50, Thermo Fisher Scientific, Madison, WI, USA) was used to identify chemical bonding and functional groups. Electrochemical impedance spectroscopy (EIS, CHI660D electrochemical workstation, CH Instruments, Inc., Austin, TX, USA) was performed over a frequency range of 0.01 Hz to 100 kHz with an amplitude of 5 mV to evaluate charge-transfer characteristics. The specific surface area and pore structure were determined by the Brunauer–Emmett–Teller (BET) method using a Micromeritics ASAP 2020 surface area analyzer (Micromeritics Instrument Corporation, Norcross, GA, USA), while pore-size distributions were calculated using the Barrett–Joyner–Halenda (BJH) method based on nitrogen adsorption–desorption isotherms.

### 2.4. Electrochemical Evaluation

The electrochemical performance of the MXene@Ge@NF anode was evaluated using CR2032-type coin cells assembled in an argon-filled glove box (O_2_ and H_2_O < 0.1 ppm). After drying, the MXene@Ge@NF electrode was gently flattened manually to improve surface planarity and facilitate handling. No controlled mechanical compression or densification treatment was applied. The electrode was subsequently punched into 12 mm disks for coin-cell assembly. Lithium metal foil was employed as the counter/reference electrode, and Celgard 2500 polypropylene membrane was used as the separator. The electrolyte consisted of 1.0 M lithium hexafluorophosphate (LiPF_6_) dissolved in ethylene carbonate (EC), diethyl carbonate (DEC), and ethyl methyl carbonate (EMC) with a volume ratio of 1:1:1. Cyclic voltammetry (CV) and electrochemical impedance spectroscopy (EIS) measurements were performed using a CHI660D electrochemical workstation. CV tests were carried out at a scan rate of 0.2 mV s^−1^ within a potential window of 0.005–3.0 V (vs. Li/Li^+^). EIS measurements were conducted by applying a 5 mV AC amplitude over a frequency range of 0.01 Hz to 100 kHz. Galvanostatic charge–discharge (GCD) tests were performed using a LAND CT2001A battery testing system within the same voltage range (0.005–3.0 V) under various current densities to evaluate cycling stability and rate performance.

## 3. Results and Discussion

### 3.1. Characterization of the Samples

**Figure 2:**
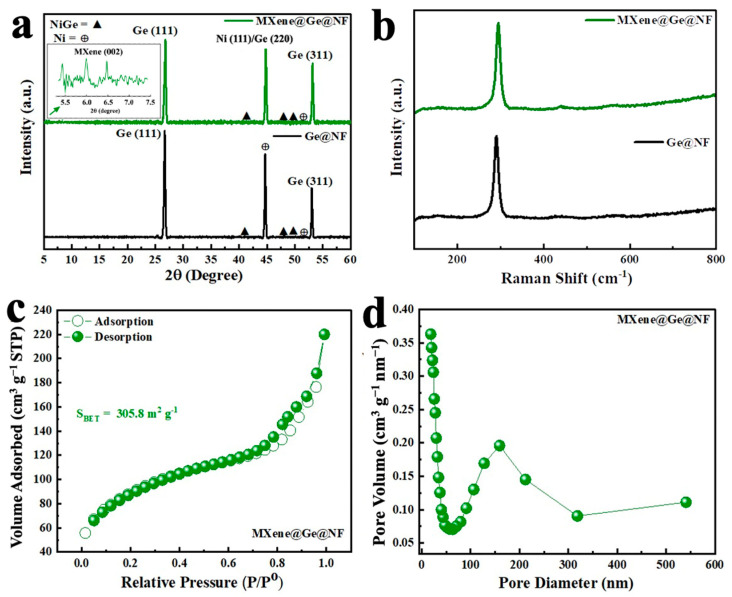
(**a**) X−ray diffraction patterns of the MXene@Ge@NF and Ge@NF composites (from top to bottom). (**b**) Raman spectra of the MXene@Ge@NF and Ge@NF composites (from top to bottom). (**c**) Nitrogen adsorption−desorption isotherms of MXene@Ge@NF composite. (**d**) The corresponding pore−size distribution of MXene@Ge@NF composite.

The crystal structure and phase composition of Ge@NF and MXene@Ge@NF were first examined by X-ray diffraction over the 2θ range of 5–60°, as shown in Figure 2a. Both electrodes exhibit the characteristic Ge (111) reflection at approximately 27.3°, while the intense feature near 44–45° contains overlapping contributions from Ni (111) and Ge (220) [32]. The reflections near 51.8° and 53.7° are assigned to Ni (200) and Ge (311), respectively, confirming the presence of crystalline Ge on the nickel-foam framework. In addition, weak reflections within approximately 40–50°, marked by triangles in Figure 2a, are consistent with a possible minor NiGe-type interfacial phase formed during annealing [33]. The MXene@Ge@NF pattern further shows a broad low-angle reflection near 6.3°, corresponding to the (002) plane of Ti_3_C_2_T_x_ MXene. Its relatively low intensity and broad profile are attributed to the limited MXene loading, few-layer morphology, and reduced long-range stacking order after exfoliation [34].

To distinguish the diffraction contribution of the nickel substrate and verify whether annealing alone produced additional crystalline phases, control XRD measurements were performed on bare nickel foam. As shown in Figure S2a, the patterns of bare nickel foam and Ge@NF were compared directly over the same 2θ range of 20–60°. The Ge@NF sample displays the characteristic Ge reflections in addition to the strong Ni-substrate peaks, and the overlap of Ni (111) and Ge (220) near 44–45° is clearly identified. Figure S2b compares bare nickel foam before and after annealing at 850 °C for 2 h under Ar. Both patterns exhibit only the characteristic Ni (111) and Ni (200) reflections, without detectable additional peaks or appreciable peak shifts, indicating that the thermal treatment alone does not generate an observable new crystalline phase in the nickel substrate [35]. Moreover, the weak reflections observed in the Ge-containing electrodes between approximately 40 and 50° are absent from both bare nickel-foam controls. Their appearance only after Ge deposition and annealing supports their assignment to a limited Ni–Ge interfacial reaction. Because these reflections are weak and no quantitative phase refinement was performed, they are described cautiously as a possible minor NiGe-type interfacial phase rather than as a dominant crystalline component.

The successful conversion of Ti_3_AlC_2_ MAX into Ti_3_C_2_T_x_ MXene is further confirmed by the XRD patterns in Figure S3a,b. Pristine Ti_3_AlC_2_ exhibits the characteristic (002), (101), (104), and (105) reflections. After selective removal of Al, the (002) reflection shifts from approximately 9° to 6.3°, indicating increased interlayer spacing associated with Al extraction and the introduction of surface terminations. The marked reduction or disappearance of the intense MAX-phase reflections further supports the successful formation of few-layer Ti_3_C_2_T_x_ MXene. Collectively, the XRD results confirm the coexistence of crystalline Ge, the metallic Ni substrate, and Ti_3_C_2_T_x_ MXene, together with reflections consistent with a possible minor NiGe-type interfacial phase. Accordingly, the electrochemical response and structural stability of MXene@Ge@NF are attributed to the combined contributions of the active Ge phase, conductive MXene network, PVP-derived carbon, three-dimensional nickel-foam scaffold, and limited Ni–Ge interfacial interaction, rather than exclusively to a Ge/MXene synergistic effect.

Raman spectroscopy was further employed to investigate the structural characteristics of the MXene component in the MXene@Ge@NF electrode. As shown in Figure 2b, the Raman spectra of Ge@NF and MXene@Ge@NF exhibit a dominant Ge-related vibration band centered at approximately ~297 cm^−1^, which originates from the Ge nanoparticles. The strong intensity of this Ge vibration, together with the contribution from the nickel foam substrate, partially overlaps and masks the weaker MXene-related Raman features in the composite electrode. To confirm the successful formation of Ti_3_C_2_T_x_ MXene, the pristine MXene Raman spectrum was additionally analyzed in the low-frequency region, as shown in Figure S4. The characteristic bands located at approximately 203, 324, and 472 cm^−1^ are attributed to Ti–C–Ti lattice vibrations, Ti–C bonding modes, and Ti–C stretching vibrations within the Ti_3_C_2_T_x_ framework, respectively [36,37]. The presence of these characteristic MXene vibrational features confirms the successful preparation of Ti_3_C_2_T_x_ nanosheets, while their weaker intensity in the composite electrode is mainly due to the strong Ge Raman response and signal overlap. The preservation of the Ti_3_C_2_T_x_ MXene framework after integration with Ge nanoparticles indicates the structural stability of the MXene component during electrode fabrication. This conductive MXene network is expected to provide additional electron-transfer pathways and contribute to the improved electrochemical performance of the MXene@Ge@NF electrode. Nitrogen adsorption–desorption measurements were conducted to evaluate the porous architecture of MXene@Ge@NF, as shown in Figure 2c,d. The isotherm exhibits a Type IV profile, indicating the presence of mesoporous structures dominated by slit-like pores [32,38]. As shown in Figure 2c, the isotherm exhibits a Type IV profile with a hysteresis loop, indicating the presence of mesoporous features. The nitrogen adsorption quantity reaches approximately 220 cm^3^ STP g^−1^ at P/P^0^ close to 1.0. The BET specific surface area and total pore volume are 305.8 m^2^ g^−1^ and 0.3403 cm^3^ g^−1^, respectively. The corresponding BJH pore-size distribution in Figure 2d shows a prominent mesopore peak at approximately 10.94 nm. This accessible mesoporous structure may facilitate electrolyte penetration and Li^+^ transport within the active composite [36,39]. Moreover, the hierarchical porous structure provides abundant electrolyte-accessible active sites and shortens the Li^+^ diffusion pathways within the electrode. The interconnected mesoporous channels also promote uniform electrolyte penetration and maintain effective ion transport during repeated lithiation/delithiation processes. These features are beneficial for achieving enhanced reversible capacity and rate capability in lithium-ion batteries.

Thermogravimetric analysis under a nitrogen atmosphere was retained to evaluate the thermal stability of the integrated MXene@Ge@NF electrode under inert conditions, as shown in Figure S5. The electrode exhibited gradual mass variation during heating, mainly associated with the removal of adsorbed species, decomposition of surface functional groups, and thermal evolution of the deposited active components [40]. However, because the large mass contribution from the nickel foam substrate and the overlapping thermal responses of Ge, Ti_3_C_2_T_x_ MXene, and PVP-derived carbon prevent independent separation of individual components, the N_2_-TGA profile was not used for quantitative composition determination. To further investigate the oxidation behavior of the active material, oxidative TGA/DTG measurements were performed on the MXene@Ge composite mechanically scraped from the nickel foam substrate, as shown in Figure S6 [41]. The oxidative profile exhibited a pronounced mass-loss process in the intermediate temperature region accompanied by a corresponding DTG peak, which was mainly associated with oxidation of carbonaceous species derived from PVP decomposition and removal of surface functional groups. The subsequent mass variation at higher temperatures was related to oxidation and transformation processes involving the inorganic components derived from Ge and Ti_3_C_2_T_x_ MXene. Due to the overlapping oxidation pathways and possible formation of intermediate oxide species during thermal oxidation, the oxidative TGA/DTG results of the final MXene@Ge composite were used to evaluate the overall oxidation behavior rather than to independently determine individual component fractions [42]. To further quantify the carbon contribution before MXene incorporation, oxidative TGA/DTG analysis was additionally performed on the annealed Ge/PVP-derived carbon intermediate scraped from the Ge@NF substrate after thermal treatment and before MXene deposition, as shown in Figure S7. The residual mass after oxidation was attributed to the Ge-derived oxide residue, and the corresponding Ge-to-GeO_2_ conversion was used together with the gravimetric Ge loading measurement to estimate the Ge and PVP-derived carbon contributions in the intermediate material. These results provided a complementary approach for evaluating the active-layer composition while avoiding the uncertainty associated with direct decomposition analysis of the final multi-component MXene@Ge composite. The combined thermal analyses confirmed the coexistence of carbonaceous and inorganic components within the MXene@Ge composite architecture.

**Figure 3:**
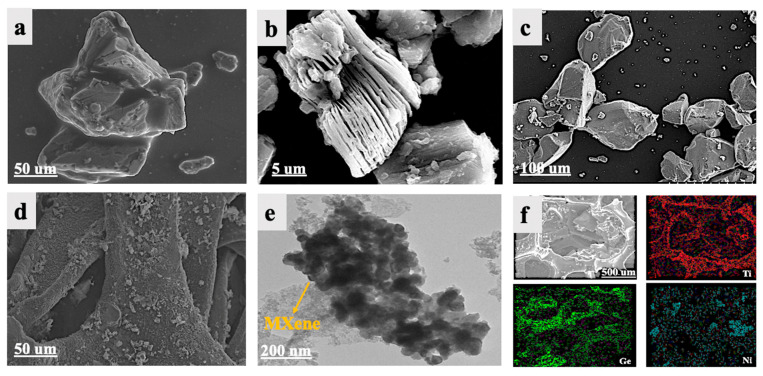
Morphological characterization of the precursor materials and the final MXene@Ge@NF electrode: (**a**) SEM image of the pristine Ti_3_AlC_2_ MAX phase; (**b**) SEM image of the exfoliated Ti_3_C_2_T_x_ MXene; (**c**) SEM image of pristine Ge particles; (**d**) SEM image of the final MXene@Ge@NF electrode; (**e**) TEM image of the MXene@Ge@NF electrode; and (**f**) the corresponding elemental maps of Ti, Ge, and Ni.

The morphology of the pristine MXene, MAX phase, and Ge nanoparticles is shown in Figure 3a–c, respectively. The MAX phase exhibits a typical layered bulk structure, while MXene displays an exfoliated sheet-like morphology, confirming successful etching and delamination [43]. The Ge precursor shows aggregated particle morphology, consistent with the supplied nanoscale powder [44]. The SEM image of nickel foam reveals a well-defined three-dimensional porous network, which provides an ideal conductive scaffold for electrode construction, as depicted in Figure S8. After deposition and thermal treatment, Ge nanoparticles are uniformly anchored on the nickel foam surface, while the porous structure of the substrate remains well preserved, indicating structural stability during processing [45]. The SEM image of the MXene@Ge@NF composite demonstrates that MXene nanosheets effectively coat the Ge-decorated nickel foam, forming a continuous conductive network, as depicted in Figure 3d. The uniform surface coverage suggests successful integration of MXene within the hierarchical framework. The TEM image further confirms the intimate contact between MXene and Ge components, as shown in Figure 3e. The contrast difference between lighter and darker regions indicates the coexistence of MXene nanosheets and Ge particles, suggesting effective interfacial coupling within the composite structure [46]. In Figure 3f, the elemental mapping clearly shows the uniform distribution of Ge, Ti, and Ni elements throughout the composite, confirming the successful construction of the MXene@Ge@NF architecture. The homogeneous elemental dispersion further supports the effective integration of all components within the hierarchical electrode.

### 3.2. Electrochemical Performances

**Figure 4:**
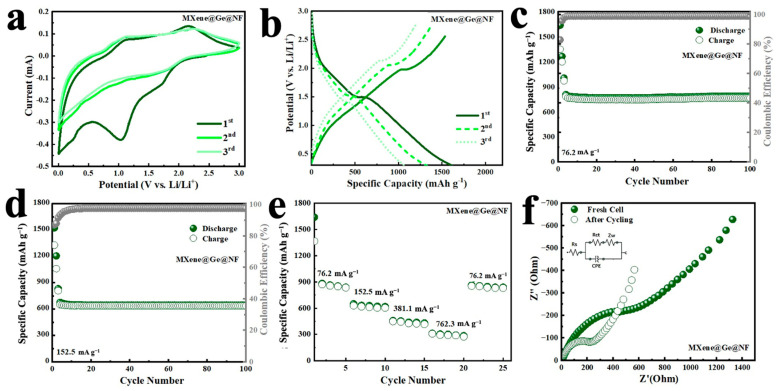
(**a**) CV results of MXene@Ge@NF electrode. (**b**) Galvanostatic charge−discharge profiles of MXene@Ge@NF electrode at 1st, 2nd and 3rd cycles at the current density of 76.2 mA g^−1^. (**c**,**d**) Electrochemical performances of MXene@Ge@NF anode during 100 cycles at current densities of 76.2 and 152.5 mA g^−1^, respectively. (**e**) Rate performance of MXene@Ge@NF anode at various current densities between 76.2 mA g^−1^ and 762.3 mA g^−1^. (**f**) EIS results of the MXene@Ge@NF anode before and after cycling.

The electrochemical behavior of the MXene@Ge@NF electrode was systematically investigated using cyclic voltammetry (CV) and galvanostatic charge–discharge (GCD) measurements, as shown in Figure 4a. The CV curves were recorded in the voltage range of 0.005–3.0 V vs. Li/Li^+^, which is consistent with Ge-based alloy anodes in lithium-ion batteries. In the first cathodic scan, a broad reduction feature appears around ~1.2 V, which is attributed to the formation of a solid electrolyte interphase (SEI) layer and initial electrolyte decomposition, while the lower potential region (~0.47 V) corresponds to the lithiation of Ge and the formation of Li–Ge alloys [47]. In the subsequent cycles, the attenuation of the SEI-related feature and the progressive overlap of the CV curves indicate stabilization of interfacial reactions and improved electrochemical reversibility [48]. From the second cycle onward, the anodic peaks at ~1.3 V and ~2.4 V are associated with the delithiation of Li–Ge alloy phases while confirming a highly reversible alloying/dealloying mechanism. Consistently, the GCD profiles of MXene@Ge@NF anode showed an initial irreversible capacity loss due to SEI formation, followed by highly overlapping charge–discharge curves in later cycles, further confirming the stabilization of the electrode/electrolyte interface and enhanced cycling reversibility after the first activation process, as shown in Figure 4b [49]. To further quantify the first-cycle irreversible capacity loss, the initial Coulombic efficiency (ICE) was calculated from the first galvanostatic charge–discharge profile. The MXene@Ge@NF electrode delivered initial discharge and charge capacities of 1639.7 and 1365.3 mAh g^−1^, respectively, at an effective current density of 76.2 mA g^−1^, corresponding to an initial Coulombic efficiency of 83.3%; the progressively overlapping subsequent galvanostatic profiles indicate improved reversibility after initial SEI formation and electrode activation [50]. These surface terminations can participate in initial interfacial reactions during lithiation, consuming additional lithium ions. Moreover, the nanosized Ge particles, porous PVP-derived carbon matrix, and exfoliated MXene nanosheets provide abundant electrochemically accessible interfaces. These components collectively influence lithium-storage behavior, while the high surface area of the composite architecture also contributes to initial interfacial reactions associated with SEI formation [51]. After the initial activation process, the conductive MXene network and three-dimensional nickel foam framework effectively facilitated electron transport, accommodated the volume variation in Ge nanoparticles, and maintained structural integrity during repeated lithiation/delithiation processes. To further evaluate the Li^+^ diffusion kinetics and understand the influence of MXene incorporation on ion-transport behavior, GITT measurements were performed for MXene@Ge@NF and Ge@NF electrodes Figure S12. The calculated apparent Li^+^ diffusion coefficients (D_Li_^+^) of the MXene@Ge@NF electrode were in the range of (2.5 × 10^−11^–8.0 × 10^−11^ cm^2^ s^−1^) during lithiation processes and (1.8 × 10^−11^–6.5 × 10^−11^ cm^2^ s^−1^) during delithiation processes. In contrast, the Ge@NF electrode exhibited lower D_Li_^+^ values of (3.0 × 10^−12^–1.5 × 10^−11^ cm^2^ s^−1^) during lithiation processes and (2.0 × 10^−12^–1.0 × 10^−11^ cm^2^ s^−1^) during delithiation processes. The higher Li^+^ diffusion coefficients and reduced polarization of MXene@Ge@NF indicate accelerated ion transport and improved reaction kinetics after MXene integration. This enhancement originates from the hierarchical MXene@Ge@NF architecture, where the conductive MXene nanosheets establish efficient electron/ion transport pathways, while the porous nickel foam scaffold promotes electrolyte penetration and buffers the mechanical stress caused by Ge volume expansion during repeated lithiation/delithiation.

The cycling performance result in Figure 4c showed that the electrode retained a reversible capacity of 789.8 mAh g^−1^ after 100 cycles at 76.2 mA g^−1^, with the Coulombic efficiency rapidly approaching approximately 100%. At the higher effective current density of 152.5 mA g^−1^, the initial discharge and charge capacities were 1525.4 and 1329.4 mAh g^−1^, respectively, corresponding to an ICE of 87.2%, while a reversible capacity of 639.5 mAh g^−1^ was maintained after 100 cycles, as depicted in Figure 4d. The recalculated rate-capability results in Figure 4e showed reversible discharge capacities of approximately 838.5, 617.5, 430.7, and 285.9 mAh g^−1^ at effective current densities of 76.2, 152.5, 381.1, and 762.3 mA g^−1^, respectively. When the current density was returned to 76.2 mA g^−1^, the capacity recovered to approximately 838.5 mAh g^−1^, indicating good rate reversibility and preservation of the conductive electrode framework. Furthermore, the higher-current cycling test in Figure S9 showed initial discharge and charge capacities of 1408.0 and 1219.7 mAh g^−1^ at 381.1 mA g^−1^, corresponding to an ICE of 86.6%, with a reversible capacity of 559.8 mAh g^−1^ retained after 100 cycles. Overall, the stable cycling and capacity recovery are attributed to the combined contributions of the MXene network, PVP-derived carbon, nickel-foam scaffold, and active Ge phase [52]. Although the capacity gradually decreased during cycling due to the irreversible structural evolution and continuous formation of interfacial films, the electrode maintained stable coulombic efficiency and considerable reversible capacity. The enhanced cycling durability can be attributed to the hierarchical Ge/C/MXene architecture, where Ge nanoparticles provide alloying-based lithium-storage sites, the PVP-derived carbon matrix contributes reversible electrochemical activity and accommodates structural variation, the MXene network facilitates electron transport and interfacial charge transfer, and the nickel foam scaffold maintains mechanical integrity during repeated lithiation/delithiation.

To further elucidate the individual contributions of the electrode components to the overall electrochemical behaviour, the cycling performances of the corresponding Ge@NF, Ti_3_C_2_T_x_ MXene, and Ge control electrodes were examined separately, as presented in Figure S10a–c. As shown in Figure S10a–c, the cycling performances of the Ge@NF, Ti_3_C_2_T_x_ MXene, and Ge control electrodes were evaluated at 100 mA g^−1^, with the specific capacities and current densities normalised using the respective total active-material mass of each electrode. The Ge@NF electrode, which contains an annealed Ge/PVP-derived carbon coating supported on nickel foam, retained a reversible capacity of 283.1 mAh g^−1^ after 100 cycles, as shown in Figure S10a. The Ti_3_C_2_T_x_ MXene control maintained 228.7 mAh g^−1^ after 100 cycles, whereas the Ge control retained 181.3 mAh g^−1^ under the same nominal current-density condition, as depicted in Figure S10b,c. All three electrodes exhibited a pronounced initial irreversible capacity loss, mainly associated with electrolyte decomposition, SEI formation, and irreversible lithium consumption, followed by progressively stabilised cycling and Coulombic efficiencies [53]. The improved capacity retention of Ge@NF relative to the isolated Ge control suggests that the PVP-derived carbon and three-dimensional nickel-foam scaffold contribute to enhanced electrical connectivity and mechanical support [54]. Meanwhile, the moderate but stable capacity of the MXene control confirmed its contribution primarily as a conductive and interfacial component rather than as the dominant lithium-storage phase [55]. These control results provide evidence that the electrochemical response of MXene@Ge@NF originates from the combined contributions of Ge, Ti_3_C_2_T_x_ MXene, PVP-derived carbon, and the nickel-foam framework. However, because the control electrodes were evaluated at a nominal current density of 100 mA g^−1^ while the MXene@Ge@NF electrode was normalized using the total active-material mass (76.2 mA g^−1^), these comparisons are considered qualitative rather than strictly quantitative. Therefore, the MXene@Ge@NF electrode should be considered as an integrated Ge/C/MXene composite system, where the electrochemical performance results from the coordinated functions of multiple components rather than from a single high-capacity active phase [56]. Therefore, the MXene@Ge@NF electrode should be considered as an integrated Ge/C/MXene composite system, where the electrochemical performance results from the coordinated functions of multiple components rather than from a single high-capacity active phase.

To further understand the interfacial charge-transfer behavior and electrochemical stability of the MXene@Ge@NF electrode, electrochemical impedance spectroscopy (EIS) measurements were performed before and after cycling. The electrochemical impedance spectra of the MXene@Ge@NF electrode showed a relatively small interfacial resistance in the fresh cell, while a slight impedance variation after cycling was associated with the formation of a stabilized electrode/electrolyte interface, as shown in Figure 4f [52]. Importantly, the relatively low impedance and preserved Warburg response suggest favorable ion transport and interfacial charge-transfer characteristics even after prolonged cycling, demonstrating the structural and electrochemical robustness of the MXene@Ge@NF electrode. The Nyquist plots of the fresh and cycled MXene@Ge@NF cells exhibit a depressed semicircle in the high-to-medium-frequency region, followed by an inclined response in the low-frequency region. The high-frequency intercept on the real axis represents the uncompensated solution and contact resistance (R_S_), whereas the diameter of the depressed semicircle reflects the overall interfacial resistance associated with overlapping surface-film and charge-transfer processes. Because only one broad semicircle is clearly resolved, the SEI resistance and charge-transfer resistance (Rct) cannot be separated reliably without overparameterizing the equivalent-circuit model. Therefore, the semicircle was described using a combined interfacial resistance (Rint). Based on the plotted spectra, the fresh cell exhibits approximate R_S_ and Rint values of 12 and 435 Ω, respectively, whereas the corresponding values after cycling are approximately 15 and 200 Ω. The relatively small variation in R_S_ indicates that the bulk electrolyte and cell-contact resistances remained comparatively stable during cycling. In contrast, the decreased Rint after cycling suggests progressive electrochemical activation of the electrode and improved interfacial charge-transfer behavior. The inclined low-frequency region is attributed to the diffusion-related impedance of lithium ions within the electrode. Therefore, the reduced interfacial resistance after cycling indicates that the integrated Ge/C/MXene architecture promotes favorable interfacial reaction kinetics by maintaining efficient electron transport pathways and stable electrode/electrolyte interactions.

After cycling, discharged cells were disassembled in an argon atmosphere inside a glove box by splitting the two sides of the metal casing with the proper cutting instrument while keeping the O_2_ and H_2_O less than 0.1 ppm. This was employed to avoid any sparking or other potentially detrimental adverse effects during the cell opening procedure. The cell’s cover was removed with great caution. The various components of the coin cell were segregated carefully. The extracted samples were not rinsed to avoid changing or altering the morphology of the electrodes. The samples were then permitted to dry within the glove box. To prevent air contamination of the electrode samples during the imaging process, they were transferred to the scanning electron microscope (SEM) using a vacuum transfer holder. After 100 cycles, the MXene@Ge@NF anode still retained a clear interconnected 3D framework, while demonstrating its excellent structural stability, as shown in Figure S11a,b. 

## 4. Conclusions

In this study, a binder-free MXene@Ge/C@NF electrode was prepared through the sequential immobilization of Ge nanoparticles within a PVP-derived carbon matrix supported on a three-dimensional nickel-foam scaffold, followed by deposition of a Ti_3_C_2_T_x_ MXene network. Structural and compositional analyses confirmed the presence of crystalline Ge, MXene, PVP-derived carbon, the nickel substrate, and a possible minor NiGe interfacial phase formed during annealing. These findings indicate that the electrochemical behaviour cannot be attributed exclusively to the interaction between Ge and MXene, but instead originates from the integrated functions of the Ge/C/MXene composite architecture. The three-dimensional nickel foam provides continuous electron-transport pathways and mechanical support, while the MXene network improves interfacial electrical connectivity and electrolyte accessibility. PVP-derived carbon and limited Ni–Ge interfacial interaction may provide additional conductive and interfacial contributions. Comparison with Ge@NF and individual component electrodes further clarifies the respective contributions of MXene, Ge, PVP-derived carbon, and the nickel-foam framework to the cycling stability and charge-transfer behaviour of the composite electrode. Using the total deposited active-material mass of Ge, Ti_3_C_2_T_x_ MXene, and PVP-derived carbon as the normalisation basis, the MXene@Ge@NF electrode retains reversible capacities of 789.8 mAh g^−1^ after 100 cycles at an effective current density of 76.2 mA g^−1^ and 639.5 mAh g^−1^ after 100 cycles at an effective current density of 152.5 mA g^−1^. The observed performance therefore arises from the combined functions of Ge, Ti_3_C_2_T_x_ MXene, PVP-derived carbon, the nickel-foam framework, and the possible minor NiGe interfacial phase. Accordingly, the main contribution of this work lies in the systematic fabrication and evaluation of a sequentially assembled, binder-free Ge/C/MXene hybrid electrode using control-electrode comparisons, structural verification, and a clearly defined active-material mass basis.

## Figures and Tables

**Table 1:** Comparison of the electrode architecture, structural characteristics, and electrochemical performance of representative Ge-based lithium-ion battery anodes.

Electrode	Substrate Material and Architecture	Ge Phase/Morphology and Structural Component	Binder-Free/Self-Supported Status	ICE (%)	Reported Cycling Performance	Ref.
Ge/C nanospheres	Conventional powder-composite electrode	Ge nanoparticles encapsulated within conductive carbon nanospheres	No; conventional composite electrode	≈64	529 mAh g^−1^ after 100 cycles at 100 mA g^−1^	[26]
Ge/GeO_2_ NPs	Conventional slurry-cast composite electrode	Mixed Ge/GeO_2_ nanoparticles	No; conventional composite electrode	69.7	211 mAh g^−1^ after 90 cycles at 100 mA g^−1^	[27]
GeO_2_/Ge/rGO-25	Graphene-supported powder-composite electrode	GeO_2_/Ge particles distributed within an rGO network	No; conventional composite electrode	≈79	≈300 mAh g^−1^ after 100 cycles at 100 mA g^−1^	[28]
Ge/RuO_2_	Conventional slurry-cast composite electrode	Ge nanoparticles dispersed within a rutile-RuO_2_ matrix	No; conventional composite electrode	56.2	471 mAh g^−1^ after 90 cycles at 100 mA g^−1^	[29]
GeO_2_-rGO-80	rGO-supported powder-composite electrode	GeO_2_ nanoparticles supported on reduced graphene oxide	No; conventional binder-containing electrode	≈68	785 mAh g^−1^ after 150 cycles at 100 mA g^−1^	[30]
Ge@C	Conventional Cu-foil-supported slurry-cast powder composite electrode	Ge nanoparticles uniformly embedded within a carbon matrix; acetylene black provides additional conductive pathways	No; conventional binder-containing electrode	≈80	780 mAh g^−1^ after 100 cycles at 100 mA g^−1^	[31]
**MXene@Ge@NF**	Three-dimensional Ni foam with a sequentially deposited active layer	Ge nanoparticles integrated with PVP-derived carbon and Ti_3_C_2_T_x_ MXene	Yes; binder-free and directly supported	83.3	789.8 mAh g^−1^ after 100 cycles at 76.2 mA g^−1^	This work

## Data Availability

The original contributions presented in this study are included in the article/supplementary material. Further inquiries can be directed to the corresponding authors.

## Supplementary Materials

The following supporting information can be downloaded at: https://www.mdpi.com/article/10.3390/nano16150969/s1, Figure S1: The preparation process of MXene@Ge@NF; Figure S2: (a) XRD patterns of bare nickel foam and Ge@NF over the 2θ range of 20–60°, showing the contributions of crystalline Ge, metallic Ni and weak reflections consistent with a minor NiGe interfacial phase. (b) XRD patterns of bare nickel foam before and after annealing at 850 °C for 2 h under Ar; Figure S3: (a,b) X−ray diffraction patterns of the MXene and MAX powder, alternatively (from top to bottom); Figure S4: Raman spectra of pristine MXene; Figure S5: TGA curves of MXene@Ge@NF; Figure S6: Oxidative TGA and corresponding DTG curves of the active MXene@Ge composite mechanically scraped from the MXene@Ge@NF electrode. The nickel foam substrate was excluded. The measurement was performed under an O_2_ atmosphere from room temperature to 800 °C at a heating rate of 10 °C min^−1^; Figure S7: Oxidative TGA and corresponding DTG curves of the annealed Ge/PVP-derived carbon intermediate mechanically scraped from the Ge@NF electrode after thermal treatment at 850 °C under Ar and before MXene deposition. The measurement was performed under an O_2_ atmosphere from room temperature to 800 °C at a heating rate of 10 °C min^−1^; Figure S8: SEM image of the pristine nickel foam; Figure S9: Cycling performance of MXene@Ge@NF anode during 100 cycles at a current density of 381.1 mA g^−1^; Figure S10: Electrochemical performances of (a) Ge@NF, (b) pristine MXene and (c) pristine Ge during 100 cycles at a current density of 100 mA g^−1^; Figure S11: (a) Digital photographs of the cycled MXene@Ge@NF anode. (b) SEM image of the MXene@Ge@NF anode after 100 cycles at 76.2 mA g^−1^; Figure S12: (a,b) GITT potential profiles of MXene@Ge@NF and Ge@NF anodes, respectively.

## References

[1] Yang Y., Liu Y., Cai J., Zhang L., Han H., Deng W., Hou H., Liu C., Zou G., Ji X. (2025). Natural minerals derived advanced materials for high performance lithium-ion and sodium-ion storage systems. Adv. Energy Mater..

[2] Khan A., Al Rashid H., Roy P.K., Chowdhury S.I., Sathi S.A. (2025). Challenges and the Way to Improve Lithium-Ion Battery Technology for Next-Generation Energy Storage. Energy Environ. Mater..

[3] Dong Y., Liu C., Rui M., Zhang X., Guan Y., Chen L., Huang Q., Wang M., Su Y., Wu F. (2025). Review on Graphite Anodes for Fast-Charging Lithium-Ion Batteries: Mechanism, Modification and Characterizations. Adv. Funct. Mater..

[4] Hu W., Pan Y., Zhong M., Wang L., Jiang W., He X. (2026). Multiscale Design Strategies for Fast-Charging Graphite Anodes in Lithium-Ion Batteries. Small.

[5] Zhang J., Zheng T. (2025). Group IVA Alloy Anodes for Sodium-Ion Rechargeable Batteries: Electrochemistry, Mechanics, and Kinetics. Batter. Supercaps.

[6] Yan G., Wang F., Liu J., Zhou H., Lei X., Wu Z., Du W., Zhong Y., Feng J., Ge Z. (2026). Contact interfaces in anodes with large volume strain for high-performance lithium-ion storage. Energy Environ. Sci..

[7] Rahman M.M., Nisar U., Abouimrane A., Belharouak I., Amin R. (2025). Valuation of anode materials for high-performance lithium batteries: From graphite to lithium metal and beyond. Electrochem. Energy Rev..

[8] Sun J., Liu W., Liang F., Liu R., Yao Y., Zou Q., Dong P., Wang S. (2026). Insight Into High Entropy Compounds: Advances, Challenges and Energy Applications. Adv. Funct. Mater..

[9] Xu G., Zhu C., Mao J., Zhao J., Li X., Cheng F. (2024). A three-dimensional Ge@C anode with a hollow and micro/nano structure for high-capacity lithium-ion battery. J. Energy Storage.

[10] Rusman E., Nulu A., Sohn K.Y. (2025). N-doped CNT assisted GeO_2_–Ge nanoparticles as a high-capacity and durable anode material for lithium-ion batteries. RSC Adv..

[11] Fang X.X., Jiang C., Yue C., Hu F. (2024). Three-Dimensional Self-Supported Ge Anode for Advanced Lithium-Ion Batteries. Chem.–A Eur. J..

[12] Zhang Z., Li L., Liu J., Guo X., Chao K., Mao C., Li G. (2024). Alleviated volume changes of germanium anode via facile chemical confinement strategy. Chem. Eng. J..

[13] Chen Y., Zou Y., Shen X., Qiu J., Lian J., Pu J., Li S., Du F.-H., Li S.-Q., Ji Z. (2022). Ge nanoparticles uniformly immobilized on 3D interconnected porous graphene frameworks as anodes for high-performance lithium-ion batteries. J. Energy Chem..

[14] Lee S.J., Kim T.W., Cho H.M., Lee M.E. (2025). One-Pot Synthesis and Optical Properties of n-Butyl-Si/Ge Nanoparticles. Nano.

[15] Yang H., Wu Y., Hu L.Y., Wang J.J., Wang F., Xu X.H. (2023). Controlled synthesis of GeSe_2_ and GeSe nanostructures induced by TBAB. Rare Met..

[16] Zhao F., Feng Y., Feng W. (2022). Germanium-based monoelemental and binary two-dimensional materials: Theoretical and experimental investigations and promising applications. InfoMat.

[17] Kim H.W., Han J. (2024). Characterization of the Ge@GeO_2_-C composite anode synthesized using a simple high-energy ball-milling process for Li-ion batteries. Korean J. Chem. Eng..

[18] Lang W., Yue C., Dang M., Wang G., Chen Y., Hu F., Liu Z., Shu J. (2023). Germanium decorated on three dimensional graphene networks as binder-free anode for Li-ion batteries. J. Power Sources.

[19] Guo S., Sun Z., Liu Y., Guo X., Feng H., Luo S., Wei C., Zheng Y., Zhang X., Kim K. (2024). Multiscale Micro-Nano Hierarchical Porous Germanium with Self-Adaptive Stress Dispersion for Highly Robust Lithium-Ion Batteries Anode. Adv. Energy Mater..

[20] Rasheed T. (2022). MXenes as an emerging class of two-dimensional materials for advanced energy storage devices. J. Mater. Chem. A.

[21] Shahzad U., Marwani H.M., Saeed M., Asiri A.M., Rahman M.M. (2023). Two-dimensional MXenes as emerging materials: A comprehensive review. ChemistrySelect.

[22] Shinde P.A., Patil A.M., Lee S., Jung E., Jun S.C. (2022). Two-dimensional MXenes for electrochemical energy storage applications. J. Mater. Chem. A.

[23] Long Y., Tao Y., Lv W., Yang Q.-H. (2024). Making 2D materials sparkle in energy storage via assembly. Acc. Chem. Res..

[24] Márquez F. (2025). MXenes in solid-state batteries: Multifunctional roles from electrodes to electrolytes and interfacial engineering. Batteries.

[25] Xu X., Zhang Y., Sun H., Zhou J., Yang F., Li H., Chen H., Chen Y., Liu Z., Qiu Z. (2021). Progress and perspective: MXene and MXene-based nanomaterials for high-performance energy storage devices. Adv. Electron. Mater..

[26] Yin L., Song J., Yang J., Huang J., Li H., Bai P., Li S., Han L. (2021). Construction of Ge/C nanospheres composite as highly efficient anode for lithium-ion batteries. J. Mater. Sci. Mater. Electron..

[27] Koo J.-H., Paek S.-M. (2021). Microwave-assisted synthesis of Ge/GeO_2_-reduced graphene oxide nanocomposite with enhanced discharge capacity for lithium-ion batteries. Nanomaterials.

[28] Arro C.R., Mohamed A.T.I., Bensalah N. (2022). Impact of Ge content on the electrochemical performance of Germanium Oxide/Germanium/reduced graphene (GeO_2_/Ge/r-GO) hybrid composite anodes for lithium-ion batteries. Mater. Today Commun..

[29] Jang J.-H., Lee M., Koo J.-H., Paek S.-M. (2022). Exfoliation and reassembly routes to a Ge/RuO_2_ nanocomposite as an anode for advanced lithium-ion batteries. Int. J. Mol. Sci..

[30] Mikhaylov A.A., Medvedev A.G., Grishanov D.A., Fazliev T.M., Chernyshev V., Mel’nik E.A., Tripol’skaya T.A., Lev O., Prikhodchenko P.V. (2023). Electrochemical behavior of reduced graphene oxide supported germanium oxide, germanium nitride, and germanium phosphide as lithium-ion battery anodes obtained from highly soluble germanium oxide. Int. J. Mol. Sci..

[31] Zhang M., Shen K., Gao M., Guo X., Zhang J., Gu H., Kong Q., Jin Z. (2024). Triblock Polymer/Acetylene Black Dual-Assisted Preparation of Ge/C Nanocomposites with Superior Lithium Storage Performance. Batter. Supercaps.

[32] Rafieerad A., Amiri A., Yan W., Eshghi H., Dhingra S. (2022). Conversion of 2D MXene to multi-low-dimensional GerMXene superlattice heterostructure. Adv. Funct. Mater..

[33] Xu F., Liu B., Ang R. (2025). Advancing Thermoelectrics in Lead-Free Rhombohedral GeTe via Interfacial Engineering With MXene. Adv. Energy Mater..

[34] Kumar S., Kumari N., Suranto C.A., Mehdi S.M.Z. (2025). 2D MXenes for biomedical applications—Recent advances, challenges, and future perspectives. J. Mater. Sci..

[35] Shi C., Zhang X., Li Z., Beyene T.T., Zheng T., Liu Y., Zhu K. (2024). Review and perspectives on preparation strategies and applications of Ti_3_C_2_ MXene for Li metal batteries/Li–S batteries. Energy Fuels.

[36] Ghosh K., Ng S., Lazar P., Padinjareveetil A.K.K., Michalička J., Pumera M. (2024). 2D Germanane-MXene Heterostructures for Cations Intercalation in Energy Storage Applications. Adv. Funct. Mater..

[37] Xiong G., Zhang G., Yang X., Feng W. (2022). MXene-Germanium Schottky Heterostructures for Ultrafast Broadband Self-Driven Photodetectors. Adv. Electron. Mater..

[38] Zhang X., Javed M.S., Ren H., Batool S., Ahmad A., Tao R., Albaqami M.D., Khan S., Wang X., Han W. (2025). Optimization of Ti_3_C_2_T_x_ performance through synergistic enhancement of GeO_x_/Ti_3_C_2_T_x_ heterostructures for ammonium-ion storage. Chem. Eng. J..

[39] Yu Z., Wang D., Lu M., Li J., Meng X., Li H. (2025). Ti_3_C_2_T_x_ MXene coated 3D ordered macroporous germanium anodes for Li-ion batteries with enhanced cycling stability and fast Li-ion mobility. Mater. Res. Bull..

[40] Jo C., Wen B., Jeong H., Park S.K., Son Y., De Volder M. (2023). Spinodal Decomposition Method for Structuring Germanium–Carbon Li-Ion Battery Anodes. ACS Nano.

[41] Martincic M., Sandoval S., Oró-Solé J., Tobías-Rossell G. (2024). Thermal stability and purity of graphene and carbon nanotubes: Key parameters for their thermogravimetric analysis (TGA). Nanomaterials.

[42] Gong K., Yin L., Pan H., Mao S., Liu L., Zhou K. (2022). Novel exploration of the flame retardant potential of bimetallic MXene in epoxy composites. Compos. Part B Eng..

[43] Mishra R.K., Avinashi S.K., Fatima Z., Singh R., Gautam C. (2025). Fabrication, surface morphology, and energy storage performance of MXene-Based materials for metal-ion batteries: A review. ACS Appl. Energy Mater..

[44] Ju Z., Qi X., Sfadia R., Wang M., Tseng E., Panchul E.C., Carter S.A., Kauzlarich S.M. (2022). Single-crystalline germanium nanocrystals via a two-step microwave-assisted colloidal synthesis from GeI4. ACS Mater. Au.

[45] Poimenidis I., Bochenek K., Martsinchyk A., Majewska K., Shuhayeu P., Milewski J., Konsolakis M. (2026). High-performance supercapacitor based on cobalt nanostructures directly grown on engineered nickel foam substrate with enhanced ion transport and cycling stability. Next Mater..

[46] Shang M., Chen X., Li B., Niu J. (2020). A fast charge/discharge and wide-temperature battery with a germanium oxide layer on a Ti3C2 MXene matrix as anode. ACS Nano.

[47] Loaiza L.C., Monconduit L., Seznec V. (2020). Si and Ge-based anode materials for Li-, Na-, and K-ion batteries: A perspective from structure to electrochemical mechanism. Small.

[48] Ren Y., Yang J., Liu Q., Bai P., Qiu H., Cui J., Zhang Y., He W. (2026). PVP-assisted construction of MOF-derived NiCo_2_O_4_@ carbon nanofiber high-conductivity networks for suppressing volume expansion toward high-performance lithium-ion battery anodes. Colloids Surf. A Physicochem. Eng. Asp..

[49] Bashirpour-bonab H. (2023). Investigation and electrochemical analysis of SEI layer formation in natural graphite anode formation process in lithium-ion battery. E-Prime-Adv. Electr. Eng. Electron. Energy.

[50] Shajid S.R., Ahmed R., Ahmed M.R., Mourshed M. (2026). MXenes in Advanced High-Performance Energy Storage Technologies: Synthesis, Properties, Applications, and Challenges. Battery Energy.

[51] Hui X., Ge X., Zhao R., Li Z., Yin L. (2020). Interface chemistry on MXene-based materials for enhanced energy storage and conversion performance. Adv. Funct. Mater..

[52] Yu Z., Gai J., Wang D., Lu M., Li J., Meng X., Li H. (2026). In-situ electrodeposition of macroporous germanium on Cu foam and coating with MXene to enhance lithium-ion battery performance. Chem. Phys. Lett..

[53] Kuo T.-R., Chuang B.-Y., Kongvarhodom C., Yougbaré S., Saukani M., Chen H.-M., Lin L.-Y. (2025). Interface-Engineered Nickel Foam Electrodes with Polydopamine and Ultraphene for High-Performance Nickel Iron Metal–Organic Framework Hybrid Energy Storage. ACS Appl. Energy Mater..

[54] Han Y., Hu J., Liu X., Liu F. (2025). Progress in surface and interface modification strategies of MXene materials for energy storage applications. Materials.

[55] Chen T., Lin Y., He N., Zhao T., Wang D., Wu Z., Guo X. (2025). Modulation of lithium plating/stripping behavior through Ge/MXene lithiophilic scaffolds. Electrochim. Acta.

[56] Yun Q., Ge Y., Chen B., Li L., Wa Q., Long H., Zhang H. (2022). Hybridization of 2D nanomaterials with 3D graphene architectures for electrochemical energy storage and conversion. Adv. Funct. Mater..

