# Supplementary material for: Ti3C2 MXene-Coated Germanium Nanoparticles on Nickel Foam for Binder-Free Lithium-Ion Battery Anodes
Source: Nanomaterials (Basel). 2026 Aug 6;16(15):969. doi: 10.3390/nano16150969 (PMC13467888; doi:10.3390/nano16150969)
Supplement: Supplementary file 1 [file nanomaterials-16-00969-s001.zip › nanomaterials-4443761-supplementary.pdf]

Supporting Information

Ti_3C_2 MXene-Coated Germanium Nanoparticles on Nickel Foam for Binder Free Lithium-Ion Battery Anodes

Junaid Aslam[1], Muhammad Arif Khan[2], Weiwei Sun[1,*] and Chao Yang [1,*]

[1] School of Environmental and Chemical Engineering, Shanghai University, Shanghai 200444, China.

[2] Department of Physics, College of Sciences, Shanghai University, Shanghai 200444, China.

*Corresponding authors: Email address: vivisun@shu.edu.cn; chaoyang@shu.edu.cn

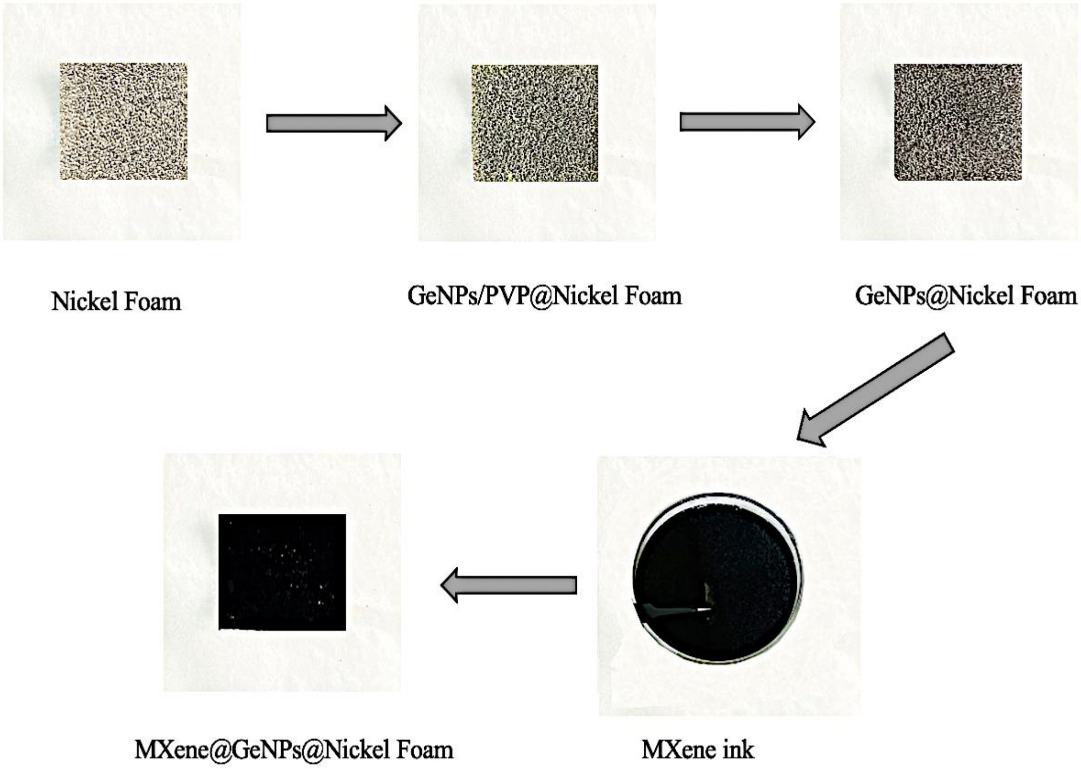

Figure S1. The preparation process of MXene@Ge@NF.

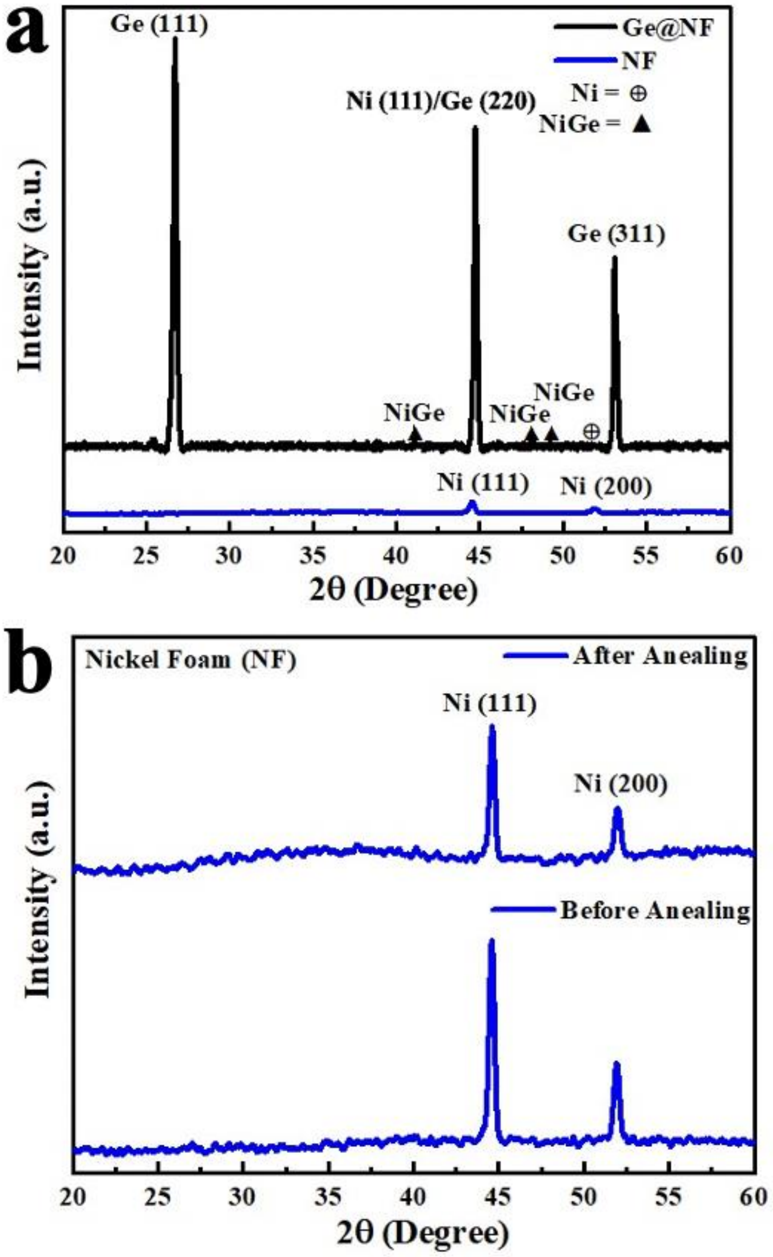

Figure S2. (a) XRD patterns of bare nickel foam and Ge@NF over the 2θ range of 20–60°, showing the contributions of crystalline Ge, metallic Ni and weak reflections consistent with a minor NiGe interfacial phase. (b) XRD patterns of bare nickel foam before and after annealing at 850 °C for 2 h under Ar.

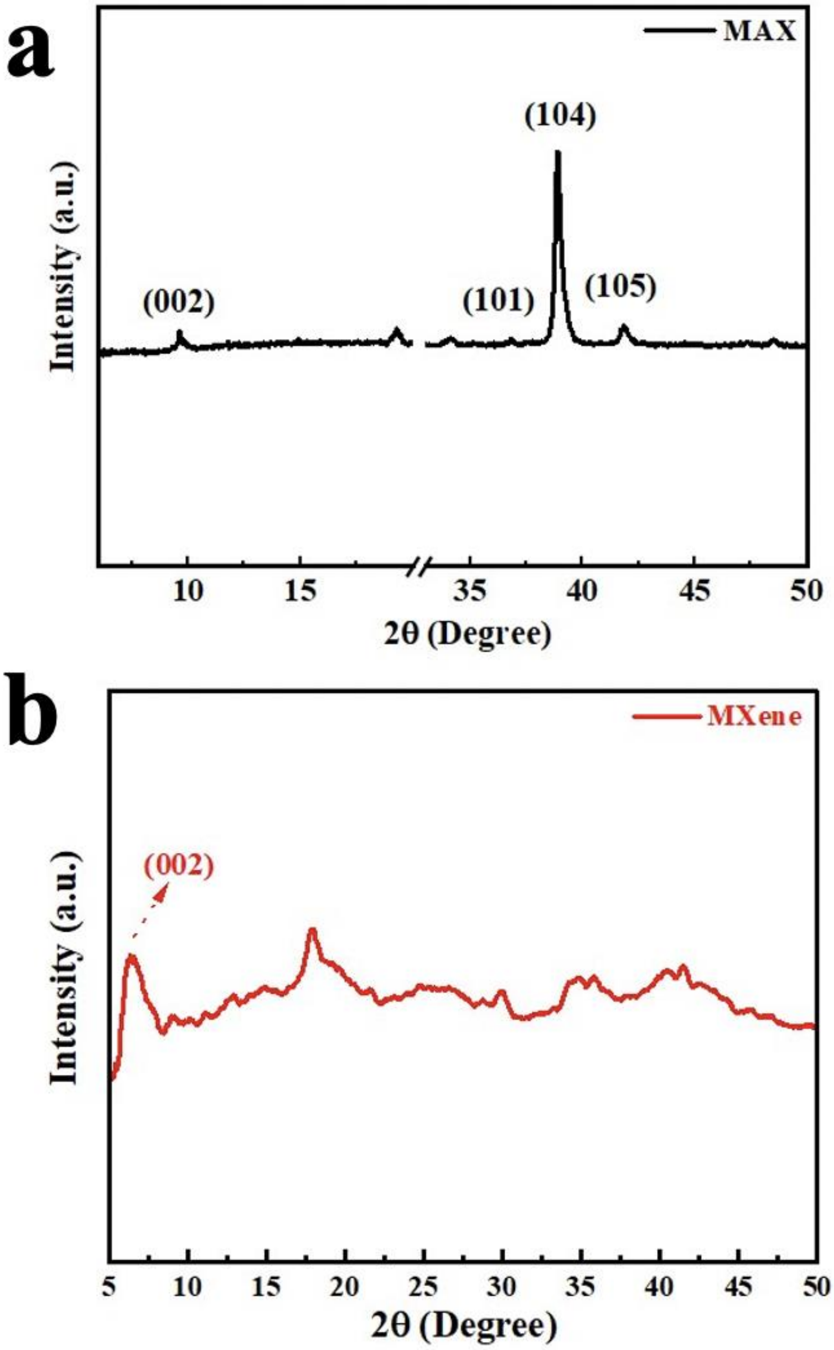

Figure S3. (a, b) X−ray diffraction patterns of the MXene and MAX powder, alternatively (from top to bottom).

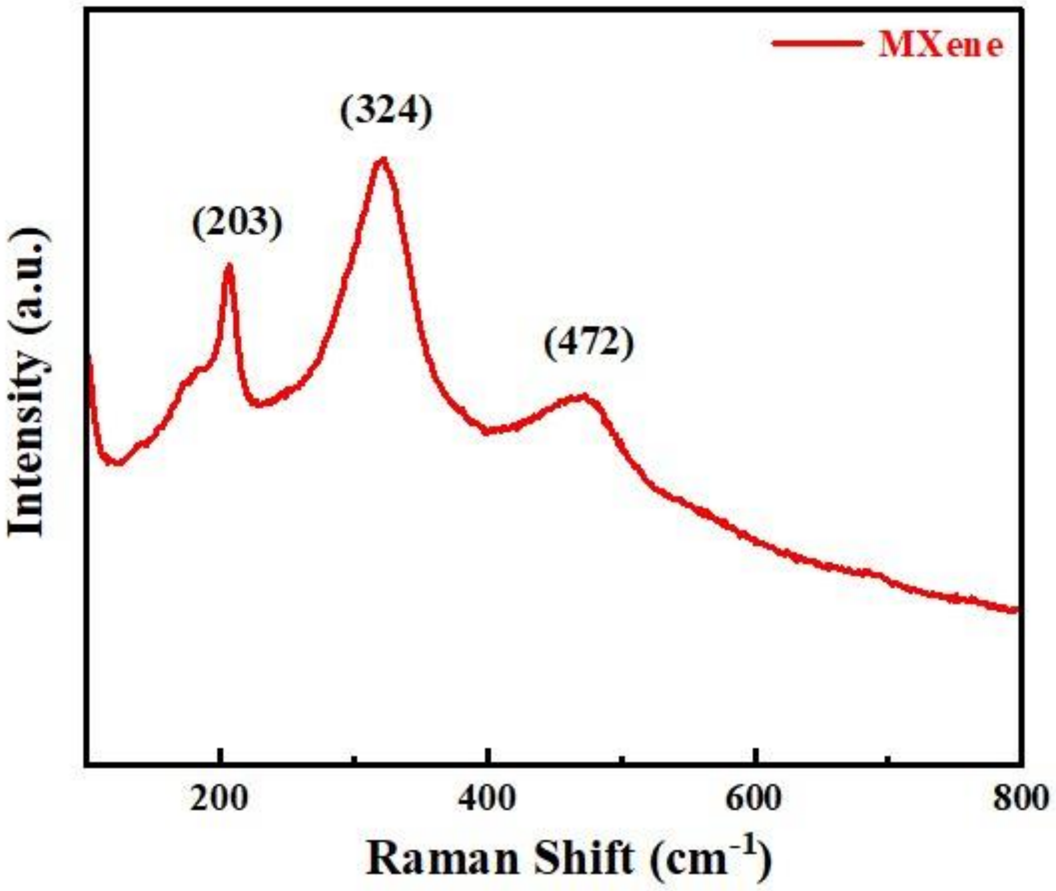

Figure S4. Raman spectra of pristine MXene.

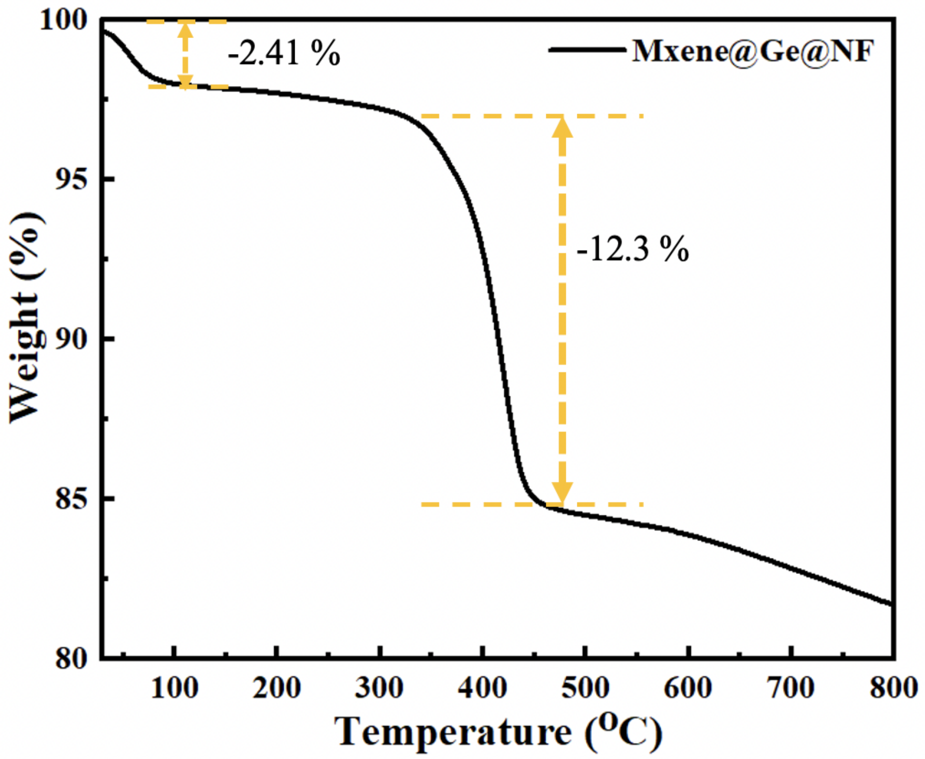

Figure S5. TGA curves of MXene@Ge@NF.

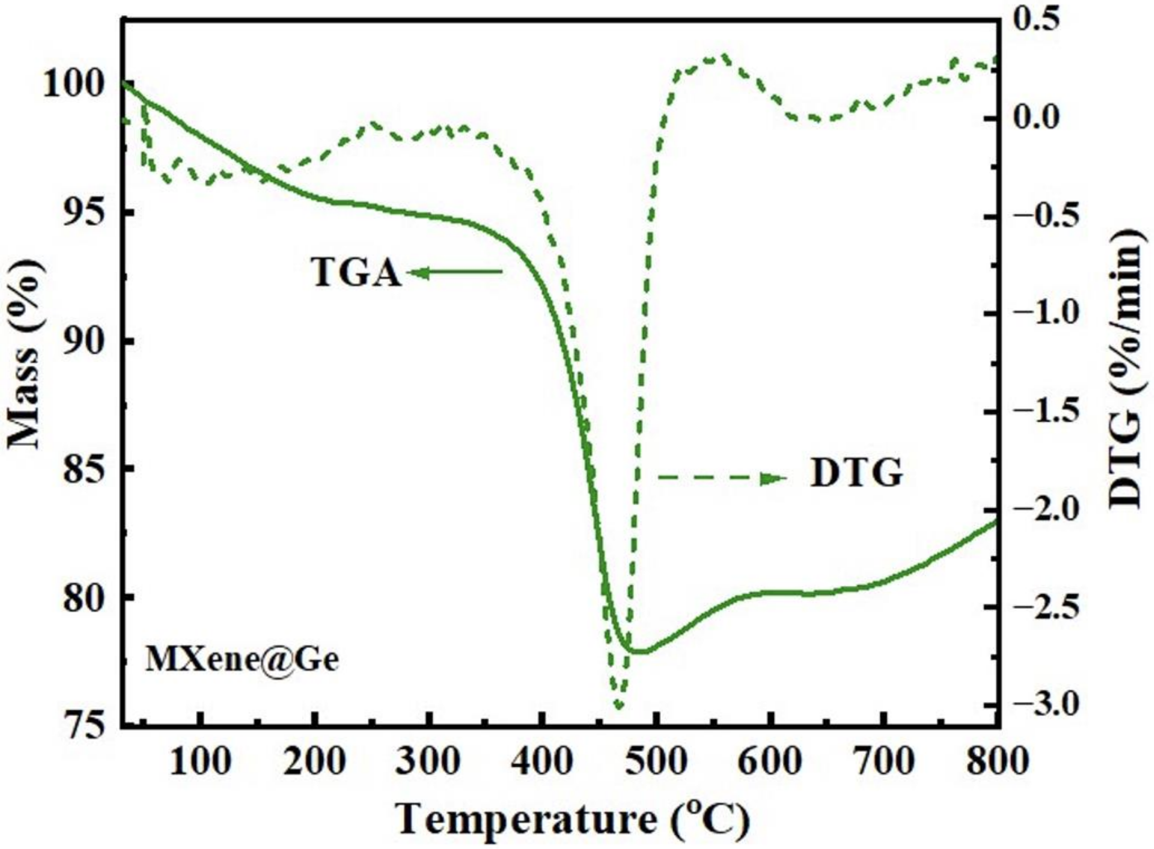

Figure S6. Oxidative TGA and corresponding DTG curves of the active MXene@Ge composite mechanically scraped from the MXene@Ge@NF electrode. The nickel foam substrate was excluded. The measurement was performed under an O_2 atmosphere from room temperature to 800 °C at a heating rate of 10 °C min^{-1}.

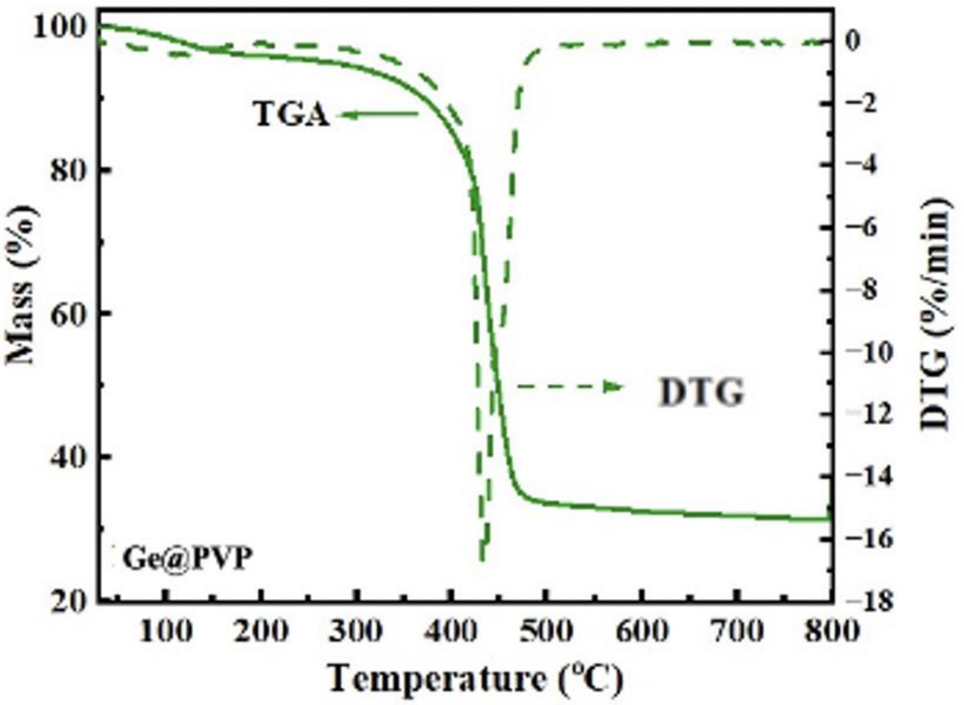

Figure S7. Oxidative TGA and corresponding DTG curves of the annealed Ge/PVP-derived carbon intermediate mechanically scraped from the Ge@NF electrode after thermal treatment at 850 °C under Ar and before MXene deposition. The measurement was performed under an O_2 atmosphere from room temperature to 800 °C at a heating rate of 10 °C min^{-1}.

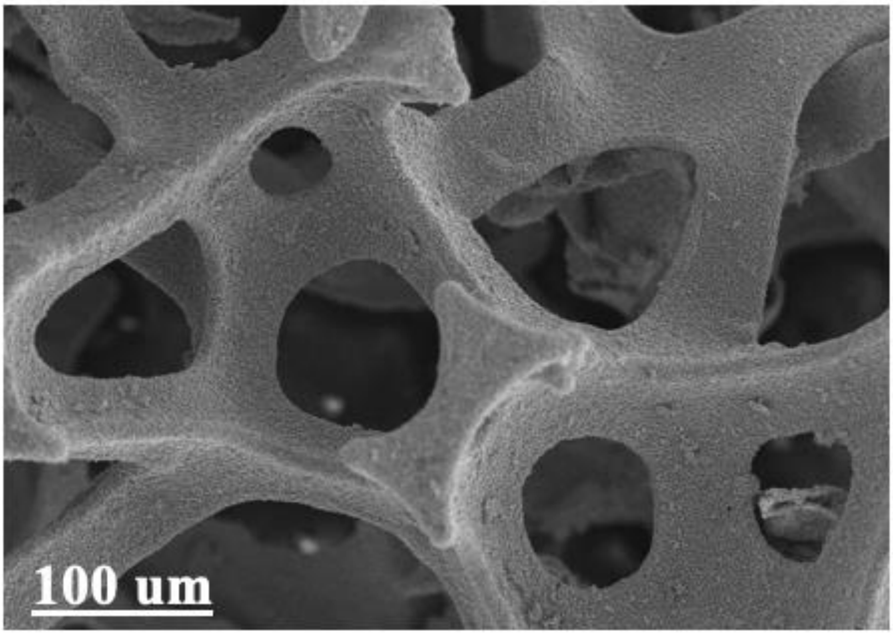

Figure S8. SEM image of the pristine nickel foam.

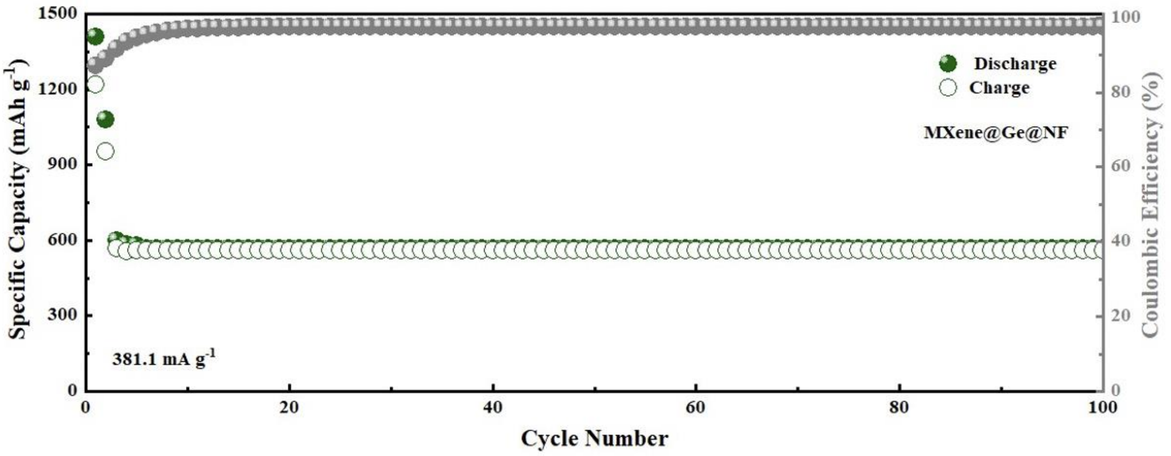

Figure S9. Cycling performance of MXene@Ge@NF anode during 100 cycles at a current density of 381.1 mA g^{-1}.

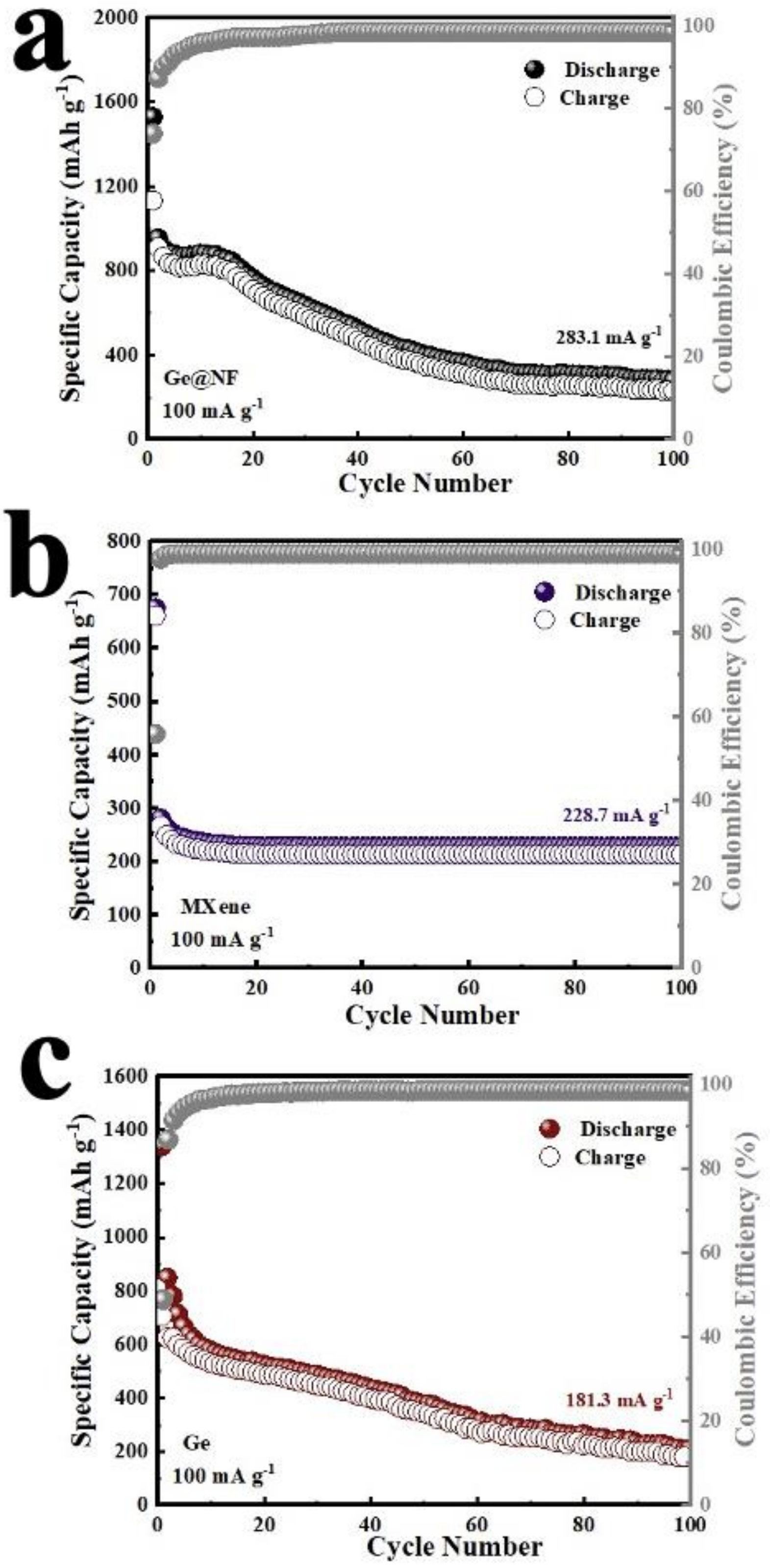

Figure S10. Electrochemical performances of (a) Ge@NF, (b) pristine MXene and (c) pristine Ge during 100 cycles at a current density of 100 mA g^{-1}.

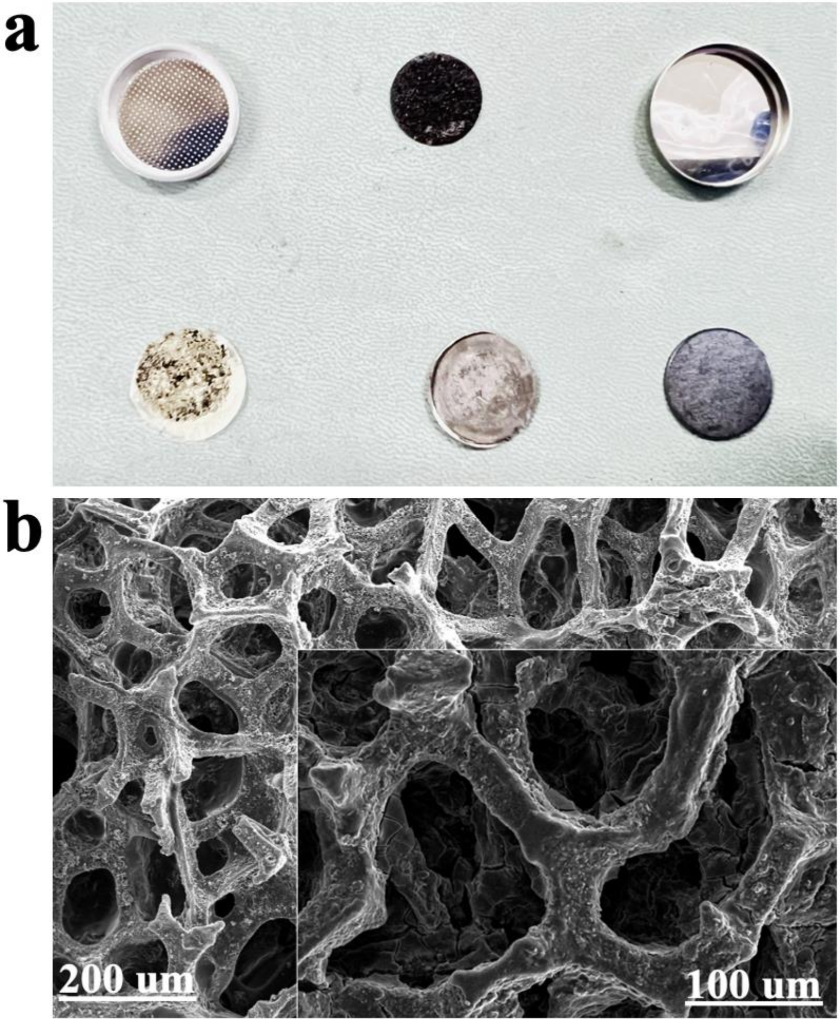

Figure S11. (a) Digital photographs of the cycled MXene@Ge@NF anode. (b) SEM image of the MXene@Ge@NF anode after 100 cycles at 76.2 mA g^{-1}.

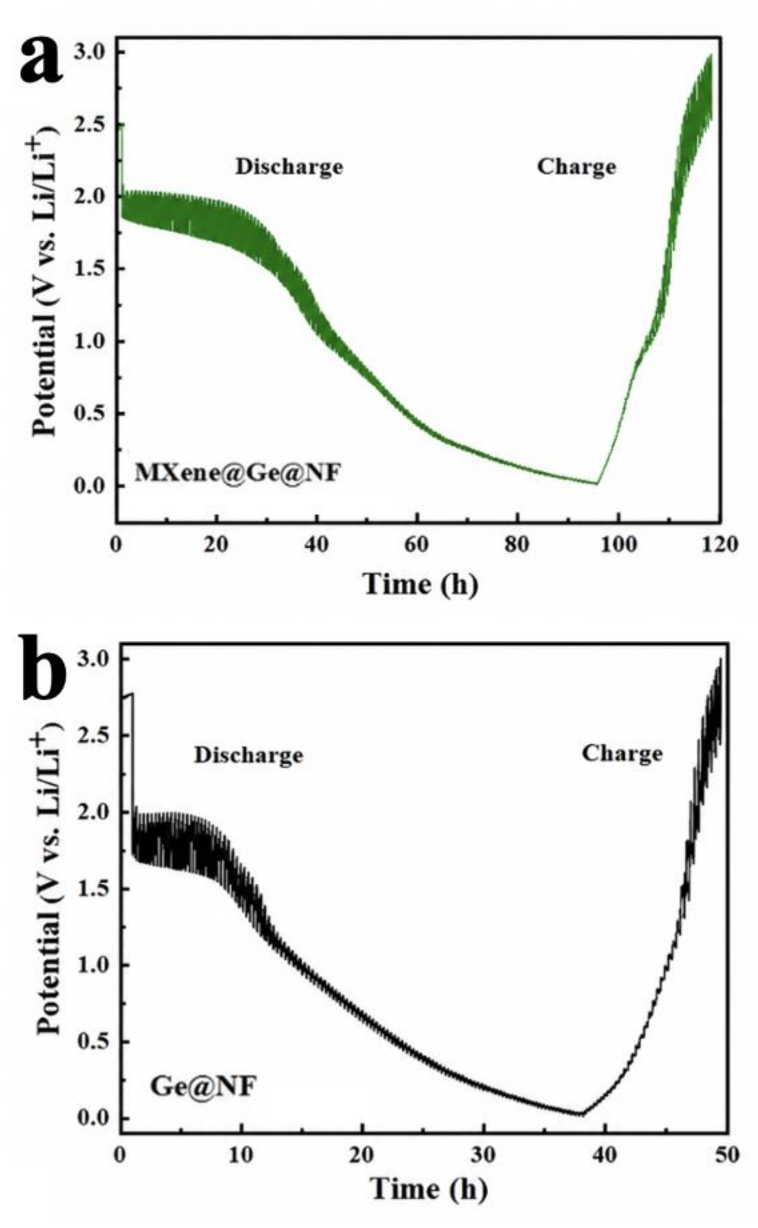

Figure S12. (a, b) GITT potential profiles of MXene@Ge@NF and Ge@NF anodes, respectively.
